# Ionic Liquid Electrolytes for Electrochemical Energy Storage Devices

**DOI:** 10.3390/ma14144000

**Published:** 2021-07-16

**Authors:** Eunhwan Kim, Juyeon Han, Seokgyu Ryu, Youngkyu Choi, Jeeyoung Yoo

**Affiliations:** School of Energy Engineering, Kyungpook National University, Daegu 41566, Korea; eunhwan93@knu.ac.kr (E.K.); gkswnus@knu.ac.kr (J.H.); waw1991@knu.ac.kr (S.R.); chokyis32@knu.ac.kr (Y.C.)

**Keywords:** ionic liquids, supercapacitor, lithium-ion battery, electrolyte

## Abstract

For decades, improvements in electrolytes and electrodes have driven the development of electrochemical energy storage devices. Generally, electrodes and electrolytes should not be developed separately due to the importance of the interaction at their interface. The energy storage ability and safety of energy storage devices are in fact determined by the arrangement of ions and electrons between the electrode and the electrolyte. In this paper, the physicochemical and electrochemical properties of lithium-ion batteries and supercapacitors using ionic liquids (ILs) as an electrolyte are reviewed. Additionally, the energy storage device ILs developed over the last decade are introduced.

## 1. Introduction

Energy storage system (ESS) and electric vehicle (EV) markets have been growing every year, and various types of energy storage devices are struggling to enter the market [1,2]. In particular, fuel cells (FCs), lithium-ion batteries (LIBs), and supercapacitors (SCs) are competing with one another in the EV market [3]. FCs have attracted a great deal of attention as energy conversion devices [4]. However, there remain difficulties in their commercialization based on the disadvantages associated with transporting, storing H_2_, and the reluctance to establish H_2_ stations [5,6,7]. Further, because of their narrow operating voltage (theoretically 1.23 V), a stacking process is essential for the application of FCs [8]. In addition, the reaction to convert hydrogen and oxygen into water is highly exothermic, thus FCs face heat management issues [9,10,11,12,13].

LIBs are considered as one of the candidates for energy storage devices owing to their high energy density and technological maturity. However, LIBs still have cost and safety issues. Because the raw materials for cathodes, such as cobalt and lithium, are produced only by a few countries, there is an unstable supply, and the materials undergo price fluctuations [14]. In addition, the electrolytes of LIBs typically include conventional binary carbonate solvents, which have high permittivity and low viscosity, and lithium salts. Li salts include lithium hexafluorophosphate (LiPF_6_), lithium tetrafluoroborate (LiBF_4_), lithium bis(trifluoromethanesulfonyl)imide (LiTFSI), etc. [15]. These electrolytes have issues in terms of poor humidity sensitivity, resulting in the formation of hydrogen fluoride (HF) [16] and the repeated formation of a solid electrolyte interphase (SEI) layer [17]. Typically, the reduction potential of organic solvents used for LIBs is 1.0 V (vs. Li^+^/Li). Thus, when an electric current is applied with Li exposed to a solution, a reaction occurs between Li and the electrolytes. The insoluble product between Li ions, anions, and solvents on the electrode surface is called the SEI layer. The growth of the SEI layer causes dendritic growth of lithium, which induces lower coulombic efficiency and threatens the life of the LIBs [18]. In addition, traditional organic solvents are not stable for a wide temperature range. For this reason, electrolytes based on organic solvents are highly volatile and flammable, which can lead to device deterioration and conflagration. Thus, the potential hazards of common organic solvent electrolytes have inspired the development of non-flammable electrolytes with thermal and electrochemical stabilities.

SCs have been proposed as an alternative energy storage device due to their high power density and extremely long lifespan. In the case of LIBs, their charge storage mechanism involves chemical reactions (reduction and oxidation), leading to a slow overall reaction. Thus, the power density of LIBs is lower than that of SCs. From this point of view, SCs are a viable substitute for devices that require a high-power density. Up to now, SCs have been regarded as an appropriate power source for electric buses that use regenerative braking as they start and stop frequently. In addition, SCs are suitable as intermittent power supplies. In this respect, they have been applied to electric trains powered by pulse power [19]. However, SCs have the critical intrinsic issue of a low energy density caused by their reaction mechanism. Thus, the development strategy of SCs is enhancing specific energy with reasonable specific power density.

The specific energy of SCs is proportional to the specific capacitance and the operating potential range [20,21]. Initially, research trends focused on the improvement of non-faradaic electric double layer (EDL) capacitance by adopting electrode active materials, which have large surface areas [17]. Thus, porous active materials, such as activated carbon (AC) and nano-structured carbon, have been studied. Additionally, pseudo-electrode materials, such as metal oxides and conducting polymers, were studied to utilize pseudo-capacitance originated from a faradaic reaction. However, these materials have some issues involving the selection of the electrolyte, cycle stability, and high cost than common SCs based on AC electrodes [22,23,24,25,26]. Other research of pseudo-capacitance has included the adoption of an electrolyte that contains redox-active couples [19,20]. Most electrolyte-adopting redox couples have a solvent limitation as these redox couples are mostly used in the form of an aqueous solution [27,28]. Hydroxide or protons are utilized as a redox couple that is generated from an aqueous solution, so the limiting voltage of aqueous redox-active electrolytes is about 0.8 V [29,30,31,32,33]. These limitations regarding active materials have motivated the improvement of the electrochemical stability window of electrolytes for high energy densities.

To overcome conventional electrolyte issues, many researchers have been focused on ionic liquids (ILs). At the early stage, ILs were studied as solvents. Generally, organic solvents used as reaction solvents in various chemical processes are highly volatile and explosive, and most of them are harmful to the human body [15]. Therefore, for developing environmentally friendly processes, many researchers are working on the development of next-generation solvents that can replace organic solvents.

ILs are a salt-like material composed of ionic bonds between cations and anions. They are in liquid state at ≤100 °C, stable at high temperatures, and have approximately zero vapor pressure [34]. Thus, ILs are called “green solvents” and have attracted considerable attention as eco-friendly solvents. In addition, ILs can dissolve various inorganic, organic, and polymeric materials and can easily change physicochemical properties, such as hydrophobicity, solubility, viscosity, and density; thus, they are also called “designer solvents” [34,35]. Thousands of syntheses are theoretically feasible using ILs and have unlimited potential as solvents. ILs exhibit various properties that existing organic solvents do not possess and have the advantages of being selected and synthesized as per the user’s purpose [36,37,38].

Because of the advantages of the ILs, their market is steadily growing. The global IL market was estimated to be US $20 million by 2015, of which the solvent and catalyst market was the largest with US $6 million, and the market for ILs is expected to grow due to the expansion of the application field, particularly in the growth of the energy storage field. The application fields of ILs can be divided into solvents and catalysts, energy storage, separation and extraction, and biorefinery, and among these the energy storage field with high growth potential was examined [39].

**Figure 1:**
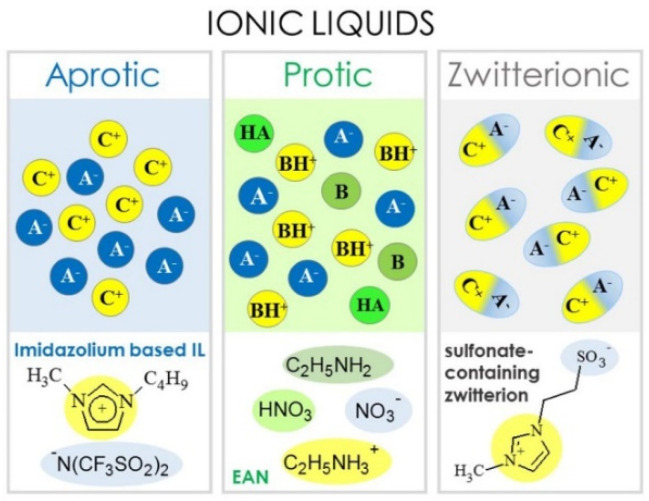
Classification of ILs: aprotic-, protic-, and zwitterionic-type. (A^−^: anion, B: base, C^+^: cation, BH^+^: Brønsted base, HA: Brønsted acid) Reprinted 2015 from [60] with permission from IOP Publishing.

ILs have satisfied the desire to develop a non-flammable electrolyte with a wide electrochemical stability window [34]. ILs consist of large organic cations and inorganic or organic anions bound by ionic bonds [35,40]. ILs show various interesting characteristics, such as non-volatility, high thermal stability, electrochemical stability, tunable polarity, basicity or acidity, and reasonable ionic conductivity [34,35]. As mentioned, ILs operate reliably within a wide electrochemical potential window of up to 6 V [36], providing high energy and power density. In addition, the tunable polarity of ILs prevents the adverse dissolution of active materials, such as water [37], and various structures of ILs are proposed by simple synthesis [38]. Because of these properties, ILs for various electrochemical devices, such as SCs, FCs, LIBs, solar cells, and actuators, have been researched [37,41,42,43,44,45,46]. ILs consist of a large organic cation, such as imidazolium, ammonium, and pyrrolidinium, with a variety of anions. Because of their large ion sizes, ILs show high viscosity, leading to a relatively low ionic conductivity. In general, in accordance with the type of cation–anion combination, ILs show a viscosity several times higher than widely used organic solvents [47]. The ionic conductivity is affected by the internal resistance, especially equivalent series resistance (ESR), limiting both the energy and power densities [48]. The energy density is decreased due to the ohmic drop induced by ESR and the power density depends on ESR, as described in Equation (1):

(1) $$P=\frac{V^{2}}{4\times \mathrm{ESR}}$$

where P represents power density and *V* is the cell operating voltage. In addition, numerous ILs exist in a solid state at room temperature, preventing actual application. To solve these issues, electrolytes produced from dissolved quaternary ammonium tetrafluoroborate in organic solvents, such as ACN or propylene carbonate (PC), were investigated [49]. The quaternary ammonium tetrafluoroborate serves as a conductive salt and was selected because of its excellent solubility, conductivity, and stability. The ionic conductivities of quaternary ammonium tetrafluoroborates contingent on their cation sizes have been reported [50]. The several quaternary ammonium tetrafluoroborates the size of tetramethylammonium and tetraethylammonium were dissolved in PC to make about 10 wt% solution and the ionic conductivity was measured at 25 °C. The cation molar conductivity decreased in the following order: tetramethylammonium > trimethylethylammonium > dimethyldimethylammonium > triethylmethylammonium > tetraethylammonium, proving conductivity is determined by ion size. Similarly, non-aqueous electrolytes containing various alkyl imidazolium salts have been reported [51]. Besides quaternary ammonium tetrafluoroborate, many other conducting salts have been reported. The ionic conductivity, specific capacitance, and thermal stability of electrolytes based on imidazolium salt, 1-ethyl-3-methylimidazolium hexafluorophosphate, and 1-ethyl-3-methylimidazolium tetrafluoroborate in organic solvents were evaluated [52]. The author prepared a cyclic and linear alkyl carbonate solvent. The cyclic carbonate has a high dielectric constant, which helps dissolution of ions, but high viscosity was observed [53]. This high viscosity resulted in low ion conductivity to hinder the ion mobility. Meanwhile, linear carbonate shows a low dielectric constant and low viscosity [54]. They investigated how changes of the dielectric constant and viscosity affect the electrolyte properties when the ILs and 1-ethyl-3-methylimidazolium salt are dissolved. They showed the similar specific capacitance in cyclic and linear carbonate when the same ILs were dissolved, demonstrating that the specific capacitance was independent of the solvent dielectric constant. The SC, which applied an organic solvent and conducting salt, kept a higher ionic conductivity; however, this type of SC limits the cell voltage to 2.6–2.9 V [55]. Cell working voltage is closely related to energy density, and the associated formula is represented as Equation (2):

(2) $$E=\frac{1}{2}CV^{2}$$

where E represents the energy density, *V* is the working voltage, and *C* is the specific capacitance of SCs. Mostly, the energy density of SCs based on a carbon electrode with organic and aqueous solvents is under 10 Wh Kg^−1^ [56,57,58,59]. Thus, SCs with organic solvents are insufficient for applications involving EVs or portable devices, which require high energy density. To achieve the higher energy density, a broad cell operating voltage is required. The cell operating voltage is related to the electrochemical stability window of the electrolyte. In addition, most organic solvents have the potential hazard of conflagration due to their high vapor pressure and flammable properties, especially at a high temperature. This characteristic of organic electrolytes necessitates careful and expensive thermal control. For these reasons, ILs are an attractive candidate for an electrolyte because of their wide electrochemical stability and remarkable thermal stability. Generally, depending on the cation chemical composition, ILs are classified as aprotic-, protic-, and zwitterion-type, as shown in Figure 1 [60].

**Figure 2:**
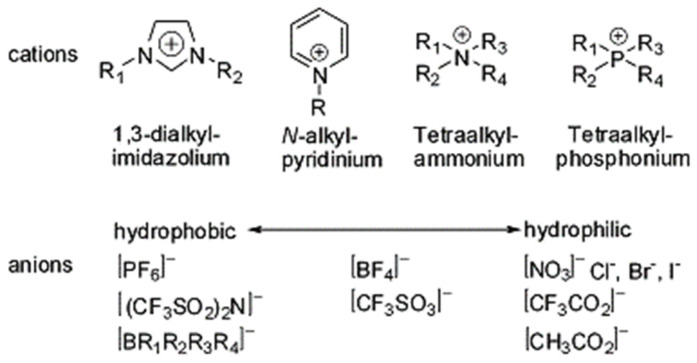
Commonly investigated cations and anions of ILs. Reprinted 2011 from [64] with permission from the Elsevier.

The aprotic-type ILs are composed solely of ions, making them appropriate for SCs and LIBs as an electrolyte. Meanwhile, protic-type ILs are produced easily with a HA and B, forming a proton migration between HA and BH^+^, and this property is suitable for fuel cells [61]. The zwitterion-type ILs contain both cations and anions that are covalently tethered [62]. The properties of ILs, including conductivity, solubility, viscosity, and melting point, are determined by their specific cation-anion combination. Hydrophobicity is associated with the anion type. As shown Figure 2, mostly imidazolium, pyrrolidinium, ammonium, sulfonium, and phosphonium cations have been investigated. Unlike the cations, anions have been investigated in a wide range. The represented anions are inorganic anions such as halides, polyatomic inorganics (PF_6_^−^, BF_4_^−^), or organic anions such as methanesulfonate (CH_3_SO_3_^−^), and acetate (CH_3_COO^−^) [63,64,65,66,67].

Recently, various LIBs and SCs applying unique characteristics of ILs have been reported. It has been demonstrated that the high solubility of ILs in polymers allows for their application to gel electrolytes with reasonable ionic conductivity. Further, volatility and flammability are reduced by adding ILs to the organic solvent electrolytes utilized in existing LIBs. In addition, ILs are effective for the prevention of poly-sulfide dissolution, resulting in improved cycle characteristics [68,69]. Furthermore, IL-based electrolytes have been reported, including poly(ionic liquid)s (PILs), and ILs blended with nanoparticles. These materials retain the properties of ILs while having new features, such as a reduced risk of leakage, increased flexibility, and a variety of desirable physical properties. Several reviews of ILs for energy storage devices have been published [70,71].

Here, we focused on an extensive review of LIBs and SCs that use various forms of ILs electrolytes. We broadly classified ILs used in LIBs and SCs. The ILs used in LIBs were classified into those used in lithium-ion batteries, lithium-sulfur (Li-S) batteries, quasi-solid-state batteries and all-solid-state batteries. The ILs used in the SCs were classified as net ionic liquids for electric double-layer capacitors, quasi-solid-state, and PIL-solid-state ILs. We then evaluated the need for ILs as an electrolyte for future research and their orientation for future research.

Also, acronyms and properties of representative ILs are provided in Table 1 and Table 2 along with descriptions of ILs used in various fields of energy storage.

## 2. Ionic Liquids for Lithium-Ion Battery Electrolytes

### 2.1. Lithium-Ion Batteries

Up to now, LIBs have typically been used with an electrolyte composed of 1 M LiPF_6_ in binary carbonate solvents or 1 M LiTFSI in ternary ether solvents. These organic electrolytes are highly volatile and flammable, leading to serious safety issues and the repeated formation of an SEI layer, which causes the dendritic growth of lithium that can lead to the short-circuiting of LIBs. Therefore, the development of electrolytes that have high thermal stability and low volatility is highly desirable for the advanced LIBs. Among the superior properties of ILs, non-flammability solves the main safety issues of LIBs.

In 2010, Zaghib proposed [EMI][TFSI] as an additive to the electrolyte for LIBs, demonstrating an increase in the thermal stability of the electrolyte [72]. Chen et al. reported that bis(fluorosulfonyl)imide lithium salt ([LiTFSI]) and the [PP_13_][TFSI] electrolyte significantly improve rate capacity and low temperature performance and are safer than conventional electrolytes [73]. In addition, inorganic alkali salts with relatively low melting points were used to improve the cycle stability and high performance of LIBs, even at high temperatures. For instance, Zhibin et al. found that dual-salt-mixed potassium bis(fluorosulfonyl)imide ([KFSI]) and [LiFSI] exhibited high ionic conductivity (10^−3^~10^−2^ S cm^−1^) from 40–150 °C [105]. In addition, designs for ionic structures and the addition of an additive to affect the properties of ILs were reported, as ILs are tunable with regard to polarity and basicity of acidity. For instance, [EMI][TFSI], [PMI][TFSI], and [BMI][TFSI] were selected as electrolytes and their electrochemical properties were investigated [70]. It was found that [EMI][TFSI] exhibited the best electrochemical performance and thermal stability. In 2009, Karna et al. synthesized several quaternary ammonium ILs based on cations with two identical ether groups and have studied quaternary ammonium-based ILs [106]. Yang et al. reported five low viscosity quaternary ammonium-based ILs [N_2_(2o1)_3_][TFSI], [(N_3_(2o1)_3_)][TFSI], [(N_4_(2o1)_3_)][TFSI]; these ILs were applied to LIBs. Among these IL electrolytes, [(N_2_(2o1)_2_(2o2)][TFSI] and [N_2_(2o1)_3_][TFSI] showed the best capacity and cycle characteristics at 0.1 C [75]. In 2014, Hirano et al. synthesized an organosilicon functionalized ammonium IL with an oligo (ethylene oxide) substituent. All ILs synthesized in this way contained large cations and had low viscosity (125–173 cP) at room temperature (RT) and showed superior thermal stability with higher decomposition temperatures (310–350 °C) and a 3.9 V–4.7 V stability window. However, they were not suitable for LIB electrolytes due to their low conductivity. Therefore, organosilicon functionalized ammonium ILs with an oligo (ethylene oxide) substituent were mixed with a commercial carbonate electrolyte to form a hybrid electrolyte. At a doping content of 30 vol%, the lithium iron phosphate (LFP) electrode/Li half-cell showed excellent reversible capacity, cycle stability, and effectively suppressed electrolyte degradation due to stable SEI formation, thus improving lithium storage performance [107]. In 2019, Hahime et al. reported that by replacing the conventional carbonate electrolyte with an RT quaternary ammonium-based electrolyte, [C_4_mpyr][TFSI], decomposition of organic solvents was suppressed and short-circuiting caused by Li dendrite formation was prevented. In the above study, the authors investigated the morphology of Li electrodeposition obtained from the [C_4_mpyr][TFSI] electrolyte and clarified the correlation between electrochemical parameters and Li deposition morphology. The surface resistance was temperature-dependent and also affected deposition polarizations and nucleation. As a result, to suppress the Li dendrite growth during cycling, the deposition polarization should be expanded during deposition while simultaneously increasing the Li diffusion coefficient of the electrolyte [76].

**Figure 3:**
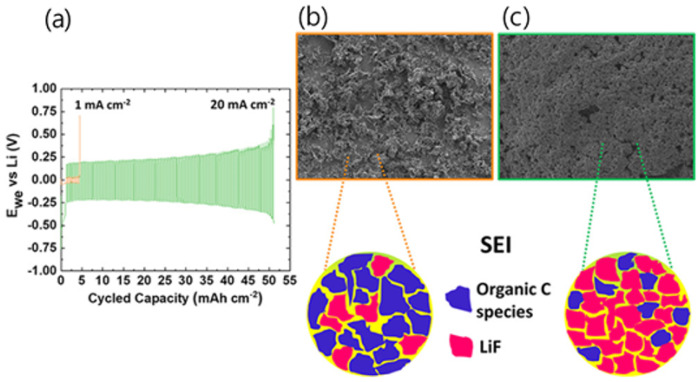
(**a**) Symmetric voltage profile at different current densities; (**b**) SEM images of deposited lithium at 1 mA cm^−2^; (**c**) SEM image of deposited lithium at 20 mA cm^−2^. Reprinted 2020 from [78] with permission from American Chemical Society.

Recently, [Pyr_14_], [Pyr_13_], and [Pyr_15_] were broadly studied for LIBs due to their high ionic conductivity and superior electrochemical performance [77,108,109]. The electrochemical performance of the LiNi_0.5_Co_0.2_Mn_0.3_O_2_ (NCM 523)/graphite full cells was also studied by Paillard et al. These studies found that [Pyr_14_][TFSI], [Pyr_14_][DCA], and [Pyr_14_][TFSAM] exhibited higher electrochemical performances than organic carbonate solvent-based electrolytes in NCM 523 and graphite full cells [74]. As the demand for energy storage devices increases, ILs with high stability, good cycle characteristics, and resistance to continuously changing temperatures have emerged. Since 2020, a method based on increasing salt concentration has been used with ILs. Qian et al. achieved high oxidation stability (>5.5 V vs. Li^+^/Li) for a graphite and Li metal anode by increasing the LiTFSI salt concentration to 4 M with [PfM_pyr_][FSI], leading to superior electrochemical cycling stability [77]. Howlett et al. reported an LIB with a high energy density system that suppressed dendritic growth despite the fast charging rate by using LiFSI salt and [C_3_mpyr][FSI]. Operating at high current densities increased coulombic efficiency up to 96% at 20 mA cm^−2^ with a 0.2 V polarization. A more detailed morphological study showed that sediment evolution remained dendrite-free and uniform with low electrode resistance (Figure 3). X-ray photoelectron microscopy (XPS), time-of-flight secondary ion mass spectrometry (ToF-SIMS), and scanning electron microscopy (SEM) surface measurements revealed that a LiF-rich SEI layer with a lack of organic components was formed. Reduced dendrite formations at high current densities are further emphasized by a 500 cycle at 10 mA cm^−2^ using a porous separator in coin cell cycling [78]. Although the conductivity of these IL-based electrolytes is lower than that of conventional carbonate electrolytes (8–12 mS cm^−1^), the overall performance of the full cell does not decrease much and is rather superior in terms of stability of the battery.

### 2.2. Lithium–Sulfur Batteries

Since 1960, Li-S batteries have been in the spotlight as next-generation LIBs due to their high theoretical capacity of 1667 mA h g^−1^ and high energy density of 2510 Wh kg^−1^. However, Li-S batteries have a number of serious problems, including the shuttle effect. In particular, the long chain of polysulfide dissolution in the electrolyte, the dendritic growth of the lithium, and spontaneous SEI layer formation results in very low cycle stability and coulombic efficiency (CE) [35,36]. Therefore, Li-S batteries have no choice but to rely on electrolytes. Numerous studies have been conducted to minimize the defects of Li-S batteries and increase their electrochemical performance [110,111,112,113].

**Figure 4:**
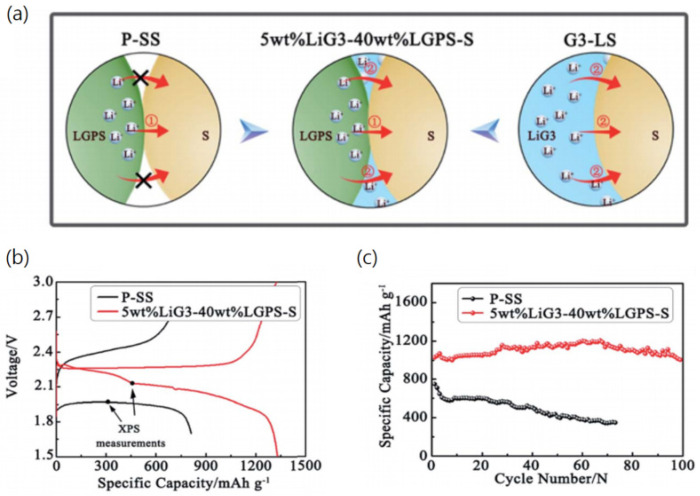
(**a**) Scheme of Li^+^ pathways in different sulfur/electrolyte interphases of a Li-S battery; (**b**) charge–discharge curves of pristine composite cathode powder (P-SS), 5 wt% LIG_3_-40 wt% LGPS-S at 50 mA cm^−2^, ambient condition; (**c**) cyclic performances at 0.2 C. Reprinted 2019 from [80] with permission from the Royal Chemical Society.

When ILs have been applied to Li-S batteries, the performance of Li-S batteries increased significantly. Yang et al. used an IL as an electrolyte for an Li–S battery and found that the ILs greatly increased the electrochemical performance and utilization of sulfur compared with organic electrolytes. Wang et al. also reported that [PP_13_]-based electrolytes improved polysulfide diffusion control and Li-metal stabilization [105]. In their paper, the [PP_13_][TFSI]/1 M LiTFSI electrolyte prohibited self-discharge. The weak Lewis acid/basic nature of [PP_13_][TFSI] induces a decrease in the coordination ability of Li^+^ with Lewis acid cations, which is expected not only to control the solubility and mobility of Li_2_S_x_ but also to inhibit the local growth of Li deposition. Peter et al. applied [C_4_mpyr][TFSI] with LiNO_3_ additives, and the electrochemical performance and SEI layer were studied and compared with conventional ether electrolytes in Li-S batteries [114]. It was found that the ILs and LiNO_3_ additive prevented the dissolution of polysulfide. Generally, the combination of ILs and additives prevents the dissolution of polysulfide, which not only increases the efficiency of Li-S batteries but also significantly reduces self-discharge. In 2019, Zuo et al. reported [LiG_3_][TFSI] as a stabilizer for Li_10_GeP_2_S_12_ (LGPS) and demonstrated a solid state Li-S battery using this formulation [80]. They confirmed that the LiG_3_ enhances the Li cationic transfer characteristic at the cathode/electrolyte interphases by adopting a solid–liquid dual phase redox reaction suppressing the shuttle effect (Figure 4a). As shown in Figure 4b,c, the [LiG_3_][TFSI] electrolyte exhibited a much higher capacity than the LGPS electrolyte and the cycle stability was also greatly improved.

### 2.3. Ionic Liquids for Lithium-Ion Batteries Using Quasi-Solid- and All-Solid-State Electrolytes

The electrolyte is a crucial factor in determining the power density, energy density, cycle stability, and safety of batteries. In general, an electrolyte based on an organic solvent is used for LIBs. This electrolyte has several stability issues, such as including flammability and poor thermal stability. A solid electrolyte can solve these problems.

In 1979, after demonstrating the solubility of poly(ethylene oxide) (PEO) against lithium salts, solid polymer electrolytes (SPEs) became widely used in LIBs [81,115,116,117]. Many researchers have since added ILs and lithium salts to escalate the ionic conductivity of PEO.

In 2017, Rhee et al. reported an IL-doped PEO-based solid electrolyte, studying the conductivity and cycle stability of the Li/SPE/LFP. The electrolyte was composed of PEO, lithium difluoro(oxalato)borate (LIFOB), and [EMI][TFSI] [116]. When 40 wt% of ILs was added at room temperature, the SPE exhibited an ionic conductivity of 0.185 × 10^−3^ S cm^−1^, with improved electrochemical stability and a first discharge capacity of 155 mA h g^−1^, which remained at 134.2 mA h g^−1^ after 50 cycles. In 2019, Wu et al. reported a flexible IL-based hybrid SPE electrolyte [81]. The hybrid SPE was fabricated with PEO, LiTFSI- [TBP][HP]. The SPE exhibited an ionic conductivity of 9.4 × 10^−4^ S cm^−1^, a wide electrochemical window over 5.0 V. The ionic conductivity of the PI solid electrolyte was 2.3 × 10^−4^ S cm^−1^ at 30 °C.

In addition, hybrid ternary polymer solid electrolyte systems composed of ILs, polymer hosts, and lithium salts were also effective in improving the electrochemical performance of LIBs. Recently, composite polymer electrolytes based on PVdF-HFP/PMMA/[BMI][BF_4_] have been reported [82]. When the PMMA content was 60%, the prepared polymer matrix absorbed [BMI][BF_4_] up to 234 wt%. This solid electrolyte maintained 96% of its initial discharge capacity after 50 cycles in the LFP/Li full cell. In 2016, Qin et al. reported a safer and more flexible battery based on a solid gel electrolyte using a PVdF-HFP/[EMI][DCA] [83]. Its conductivity was found to be 6 × 10^−4^ S cm^−1^ and it maintained a stable composition up to 300 °C. In 2021, Xu et al. reported improvements in ionic conductivity up to 2.1 using a PVdF-HFP/[EMI][TFSI]/rGO-PEG-NH_2_ (covalent linked 2,2″-(ethylenedioxy) bis (ethylamine) to reduced graphene oxide)/LiTFSI gel polymer electrolyte [84]. The fast lithium-ion transfer network of this gel polymer electrolyte showed a lithium ion transfer number of 0.45 and it was stable up to 5 V. In addition, PVdF-HFP/[EMI][TFSI]/rGO-PEG-NH_2_/LiTFSI-adopted LIBs maintained their capacity of 88% after 80 cycles.

## 3. Ionic Liquids for Supercapacitor Electrolytes

### 3.1. Net Ionic Liquid for Electric Double-Layer Capacitor

**Figure 5:**
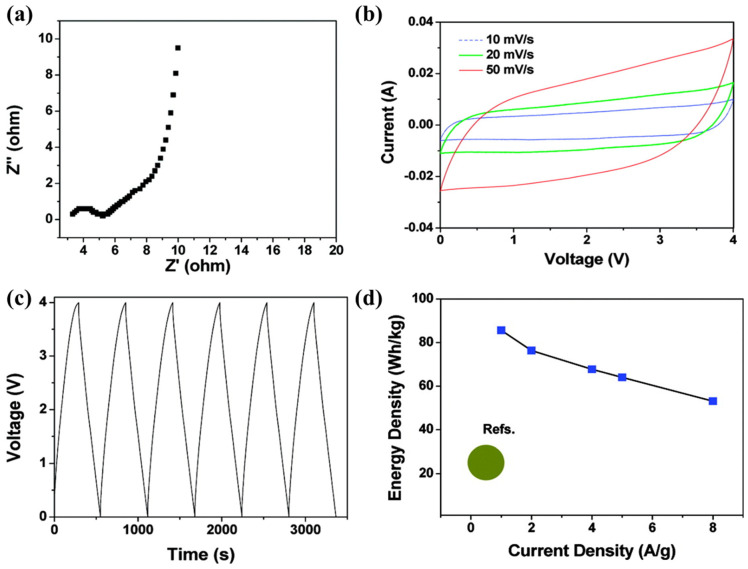
Electrochemical characteristics of graphene electrode using [EMI][BF_4_]. (**a**) Nyquist plot; (**b**) cyclic voltammograms; (**c**) galvanostatic charge−discharge profile at 1 A g^−1^; (**d**) Ragone plot compared to previous reports [121,122,123,124]. Reprinted 2010 from [101] with permission from the American Chemical Society.

Generally, imidazolium-type ILs were selected for their relatively low viscosity and high ionic conductivity [85,118]. Meanwhile, pyrrolidinium-type ILs showed a wide electrochemical stability window [117]. Vera Lockett et al. investigated the differential capacitance based on the imidazolium electric double-layer capacitor [119]. Three different imidazolium-based ILs were prepared: [EMI][Cl], [BMI][Cl], and 1-methyl-3-hexylimidazolium [HMI][Cl]. The cell was configured with a glassy carbon electrode, a Ag/AgCl reference electrode, and a Pt counter electrode, and then cyclic voltammograms and impedance spectroscopy were used to investigate the electrode/electrolyte interface. The differential capacitance was increased when the size of imidazolium cations was smaller: in the order [HMI][Cl] < [BMI][Cl] < [EMI][Cl]. This result was related to the thickness of the double-layer, showing the thinner double-layer was obtained when smaller imidazolium cations were applied. Similarly, Bettini et al. proposed a film SC based on a carbon material nanostructure (ns-C) electrode and ILs [120]. The electrolyte was varied with respect to cations, such as [BMI], [Pyr_14_], 1-dodecyl-3-methylimidazolium ([C_12_MI]), and [EMI], while the use of [FSI] anions remained constant. The ionic conductivity was increased depending on the size of the cation in the following order: [C_12_MI] < [Pyr_14_] < [BMI] < [EMI]. The SCs adopting [BMI][FSI] showed 75 F g^−1^, which was the highest specific capacitance, despite having the second-highest ionic conductivity. This implies that [BMI][FSI] has chemical affinity with ns-C. Norihisa Handa et al. suggested that the electric double-layer capacitors (EDLCs) applied to [EMI][FSI] as an electrolyte exhibited remarkable rate durability at 2 V. After 10,000 cycles, the SC maintained over 90% of the initial specific capacitance [86]. Chenguang Liu et al. displayed an SC with an ultrahigh energy density of 85.6 Wh kg^−1^ at a current density of 1 A g^−1^, as shown in Figure 5 [101]. They adopted the graphene-based electrode because of its high specific capacitance and applied the [EMI][BF_4_] as an electrolyte because of its wide electrochemical stability window (>4 V). Coupled with an electrode having high specific capacitance and an electrolyte with a broad electrochemical stability window, the highest specific energy density was achieved among carbon-electrode-based EDLCs.

The pyrrolidinium cations were investigated for their broad electrochemical stability window. A. Balducci et al. proposed the symmetric-type SC, which applied [Pyr_14_][TFSI] as an electrolyte [87]. This SC showed an operating voltage of 3.5 V, and after 40,000 cycles the SCs showed a specific energy density of 31 Wh kg^−1^ and a power density of 8.6 KW kg^−1^ at 60 °C. Because of the high working voltage, the value of the energy density was higher than the commercially achieved ACN-based EDLCs. However, due to the relatively lower ionic conductivity of ILs, the specific power density was decreased compared to SCs with applied organic electrolytes. C. Largeot et al. introduced high-temperature operating SCs based on the [Pyr_14_][TFSI] electrolyte and microporous carbide-derived carbon (CDCs) electrodes [125]. Because of the excellent thermal stability of ILs, IL-based SCs are capable of operating at a high temperature above 70 °C, which is impossible for common organic electrolytes. In addition, most pyrrolidinium-based ILs maintain a quasi-solid state at room temperature; thus, a high operating temperature is required to maintain the liquid phase and high ionic conductivity. These SCs showed a specific capacitance of 130 F g^−1^.

The electrochemical performance, in accordance with the correlation between the pore size of the electrode and the ion size of the electrolyte, has been studied [126,127,128,129]. Celine Largeot et al. presented insight between the pore size of an electrode and the ion size of an electrolyte [88]. The author tailored the pore size of the electrode for [EMI][TFSI]. Because [EMI] and [TFSI] have a roughly equal ion size (~0.7 nm), a design consisting of the same pore size of the anode and cathode is possible. CDCs were adapted as an electrode because the pore size is adjusted above or below ion size of the electrolyte. CDCs are prepared by high-temperature extraction of non-metals from a carbide precursor. The vacuum decomposition and high-temperature chlorination method is the common way to fabricate CDCs. Celine Largeot et al. fabricated CDCs via the high-temperature chlorination method. The pore size was controlled by chlorination for 3 h at a temperature range from 400 °C to 1000 °C. The pore size increased in accordance with the chlorination temperature in the range of 0.65–1.1 nm. The average pore size depending on the chlorination temperature is represented in Table 3.

**Figure 6:**
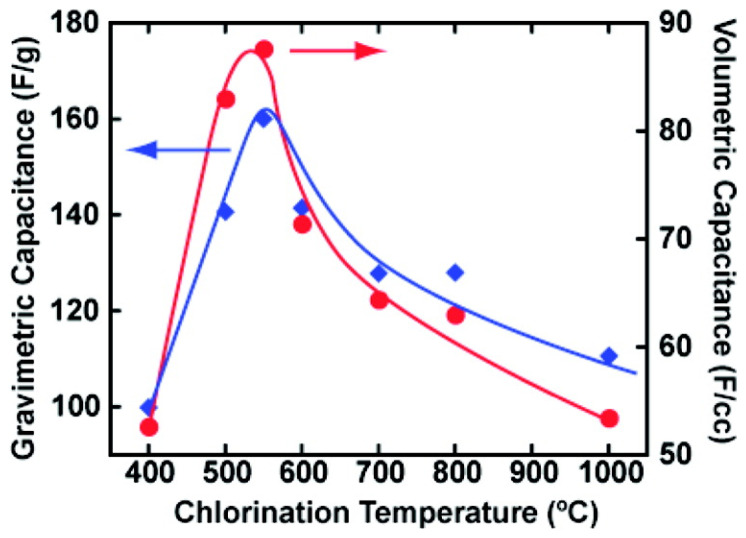
Specific gravimetric (F g^−1^) and volumetric (F cm^−3^) capacitances change depending on the chlorination temperature for CDC electrodes tested in neat [EMI][TFSI] electrolytes at 60 °C. Reprinted 2008 from [88] with permission from the American Chemical Society.

As shown in Figure 6, the x-axis implies the pore size depended on the chlorination temperature. The maximum specific gravimetric or volumetric capacitances were achieved at a 600 °C chlorination temperature. At 600 °C, the average pore width was 0.72 nm, which is very close to the ion size of [EMI][TFSI]. In this case, there is no space available for more than one ion per pore, which is implied with the ion adsorbed on both pore walls (the distance of pore and pore). They demonstrated that when the pore size was close to the ion size the ion adsorption was achieved in the most efficient way by minimizing the free space available. At under 600 °C, when the pore size of the CDCs was smaller than the ion size of [EMI][TFSI], the specific capacitance was decreased. Especially, at 400 °C, the specific gravimetric or volumetric capacitance hugely decreased because the pore size was too small to access the ions. Furthermore, above 600 °C the pore size was bigger than the ion size, and the specific capacitance was decreased. As the pore size increased, with the space for one ion per pore still free, the distance between the pore walls and the center of the ions increased, which led to lower volumetric capacitance.

In 2011, Soavi et al. reported the role of the carbon electrode and electrolyte chemistry in SCs based on the ILs [130]. The capacitive response is regulated by the carbon porosity and IL properties. In their report, the authors analyzed the cases where the pore size was wider than the size of the ion, the pore size was similar than the size of the ion, and the pore size was smaller than the ion size. When the pore was close to the IL size, the lack of electrolytes caused the high electrode polarization required for EDL charging, limiting the charge storage capacity. Meanwhile, when the pore size was wider than the ion size, an electric double-layer was easily formed because accessible ions increased with increasing pore size. At this point, properties such as conductivity, viscosity, and solvent polarity of ammonium or pyrrolidinium-based ILs such as [Pyr_14_][TFSI] and [Pyr_14_][FAP] had little effect on electrode response properties. Hence, the pore-to-ion size affects EDLC performance rather than the bulk properties of ILs. Furthermore, the density of ILs, especially [Pyr_14_][TFSI] with at least a value of 1.4 g cm^−3^, greatly contributed to the total SC weight. They insisted a sufficient amount of electrolytes is required to avoid inadequate electrolytes in carbon pores; thus, the total weight would be higher than that calculated using only the electrode material loadings.

**Figure 7:**
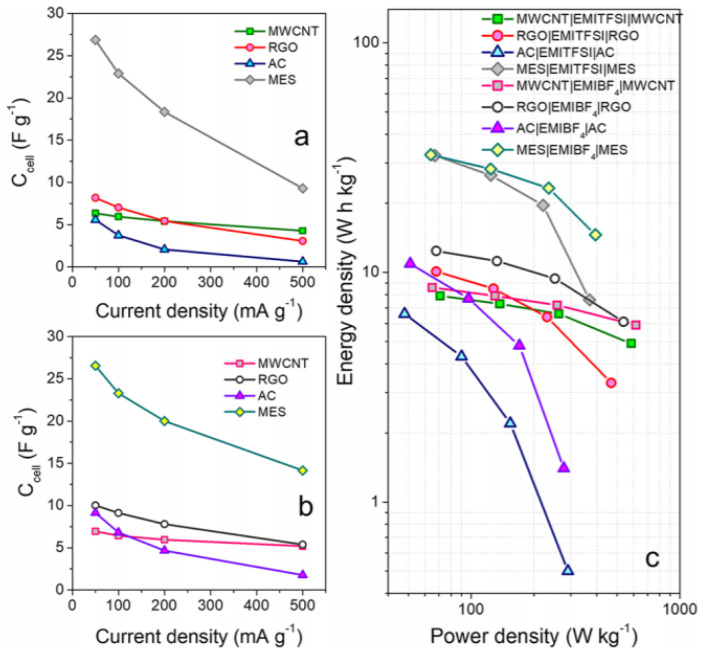
Specific cell capacitance for the EDLCs with different porous carbon electrodes and with (**a**) EMITFSI and (**b**) ${\mathrm{EMIBF}}_{4}$ as electrolytes; (**c**) Ragone plot for all evaluated EDLCs. Reprinted 2020 from [131] with permission from the American Chemical Society.

Recently, Ortega et al. investigated ion–electrode compatibility based on four different carbon-based electrodes, such as AC, mesoporous carbon (MES), multi-walled carbon nanotubes (MWCNTs), and reduced graphene oxide (rGO) [131]. They insisted that considering only pore and ion size for choosing the carbon electrode is insufficient. The materials with a high specific surface area showed specific capacitance and energy density, but in terms of power density, rGO or MWCNT, which have a more open surface, were more desirable (Figure 7). Furthermore, they reported the interaction between the electrode surface and ion, suggesting MWCNT and rGO were desirable for the negative electrode, and AC and MES for the positive electrode.

### 3.2. Other Approaches of Ionic Liquids as a Liquid Electrolyte

**Figure 8:**
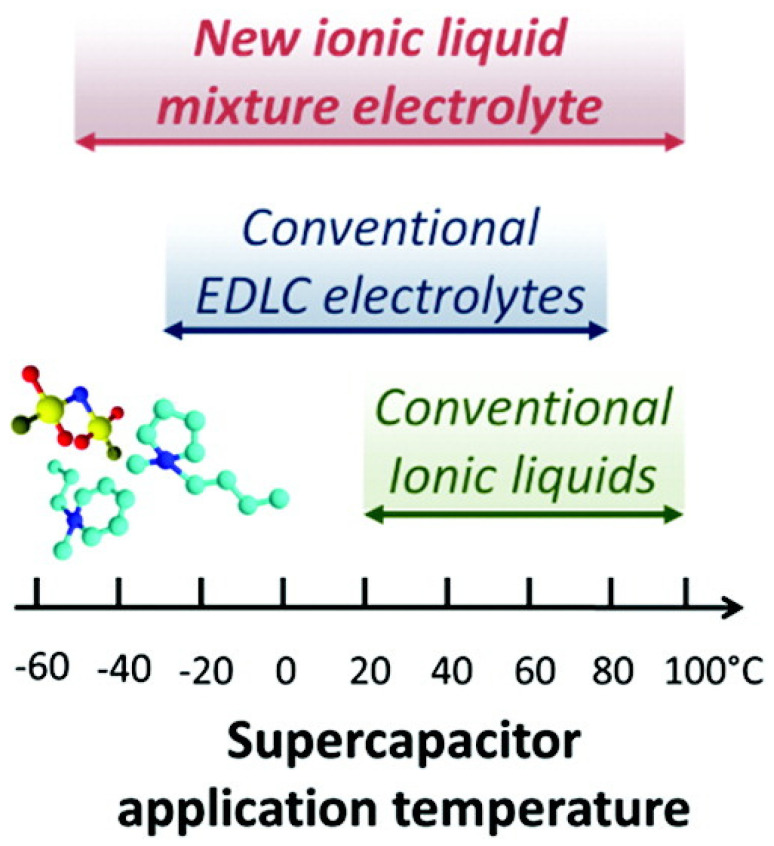
Extended operating temperature range of SCs based on eutectic ILs mixture. Reprinted 2011 from [89] with permission from the American Chemical Society.

Since ILs typically have a melting point over 0 °C, eutectic IL mixtures have been studied to reduce the melting temperature and enhance the liquid range (Figure 8). A eutectic mixture is defined as a mixture of organic salt and other compounds (urea or chlorine) that inhibit the crystallization of one another at certain ratios, resulting in a decrease in melting point [132]. It was demonstrated that the crystallization process is mainly affected by anions [88,130,131,133,134,135]. Simon et al. were able to extend the temperature range from −50 °C to 100 °C by mixing [PIP_13_][FSI] and [Pyr_14_][FSI] [89].

**Figure 9:**
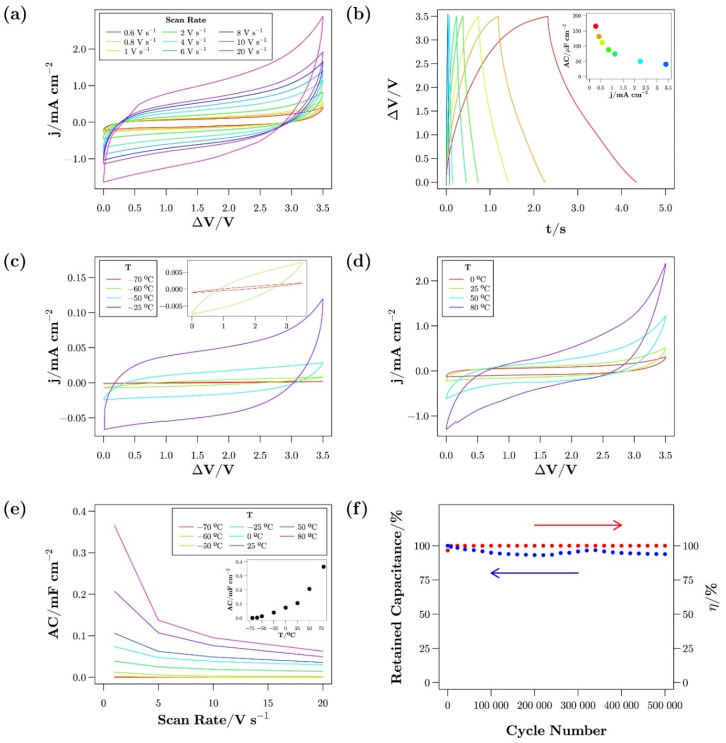
Electrochemical characteristics of Ni-foam SCs at different operating temperatures. (**a**) Cyclic voltammogram with different scan rate. (**b**) Galvanostatic charge–discharge profile. The inset shows the AC as a function of the current density. (**c**) Cyclic voltammogram at low temperatures. (**d**) Cyclic voltammogram at medium–high temperatures. (**e**) AC as a function of the scan rate obtained at different temperatures ranging from −70 °C up to 80 °C. (**f**) Cycling stability (blue points) and coulombic efficiency (red points) at ambient condition. Reprinted 2018 from [91] with permission from Elsevier.

To prevent an ordered arrangement and crystallization, the appropriate combination of cations with the same anion is essential. This combination restrains the formation of a lattice. In addition, SCs fabricated with exohedral nanostructured carbon electrode and eutectic IL mixtures as an electrolyte demonstrated a wide electrochemical stability window (>3.7 V) and a very high charge/discharge scan rate of up to 20 V s^−1^. Timperman et al. proposed a protic eutectic IL mixture of pyrrolidinium ([Pyr]) nitrate ([NO_3_]) and [Pyr][TFSI] [90]. The eutectic mixture maintained a liquid phase at 60 °C to 100 °C, whereas individual ILs existed in a solid state at room temperature. The SC fabricated with binary protic IL electrolytes and AC electrode exhibited a capacitance of 148 F g^−1^ at 2 V. Newell et al. reported the use of an [EMI][TFSI] and [PMPyrr][TFSI] eutectic IL mixture as an electrolyte for EDL capacitors [91]. The eutectic IL-based SC exhibited a specific capacitance of 5 μF cm^−^^2^ (−70 °C) and 100 μF cm^−^^2^ (80 °C) at 20 V s^−^^1^. Besides, suggested SC showed excellent specific capacitance retention up to 500,000 cycles at an operating voltage of 3.5 V (Figure 9).

A new strategy for producing eutectic IL mixtures was proposed because of the expensive cost of ILs. Fletcher et al. presented ternary mixtures composed of ILs and a polar solvent [92], including ternary mixtures of sulfolane, 3-methyl sulfolane, and butyltrimethylammonium ([BTM])[TFSI]. Sulfolane was chosen for its high thermal stability and ease of mixing with ILs. The high freezing point of sulfolane was decreased by mixing it with 3-methyl sulfolane. By adding a 60/40 eutectic mixture to [BTM][TFSI], the ionic conductivity was enhanced from 2.1 to 5.0 mS cm^−1^ at room temperature. The SC based on the AC electrode and ternary mixture electrolyte showed a wide electrochemical stability window up to 7 V.

Another approach of using ILs is as a redox-active electrolyte. The redox-active electrolyte is regarded as a promising electrolyte for enhancing the energy density of SCs. The redox-active couples dissolve in the electrolyte and undergo a faradaic reaction at the electrode/electrolyte interface, contributing the pseudo capacitance [136,137,138,139]. Since such redox couples show excellent solubility in aqueous electrolytes, many studies based on aqueous electrolytes have been reported. However, the aqueous electrolytes decompose at 1.23 V, limiting the cell operating voltage. To enhance the energy density, a relatively high operating cell voltage is required; thus, numerous IL-based redox-active electrolytes have been studied. The halide redox couple, particularly iodine/iodide or bromide/bromine, has been investigated. When 5 wt% of [EMI] iodide ([I]) was added to [EMI][BF_4_], the specific capacitance was about 50% higher than when only [EMI][BF_4_] was applied to the SC [140]. Similarly, Yamazaki et al. discovered that using [EMI][Br]/[EMI][BF_4_] as an electrolyte presented a higher specific capacitance than without bromide [141]. However, the weight ratio is inappropriate for representing the number of ions in the [EMI][I]/[EMI][BF_4_] mixture because the EDL capacitance is derived from the number of ions. Thus, our team proposed a redox-active electrolyte containing an equal number of ions [142]. The proposed redox-active electrolyte consisted of [EMI][TFSI] and [EMI][X] (X = Br, I) as redox-active couples. The SCs with a 0.12 mole fraction of [EMI][I] showed 175.6 Wh kg^−1^ and 4994 W kg^−1^ at 1 A g^−1^, showing up to 5000 cycles.

Xie et al. proposed an SC based on redox-active ILs by modifying the cations or anions with ferrocene to minimize self-discharge [127]. The modified IL-based SC exhibited an operating voltage of 2.5 V and an energy density of 13.2 Wh kg^−1^, which was 83% higher than that of the unmodified IL-based SCs.

**Figure 10:**
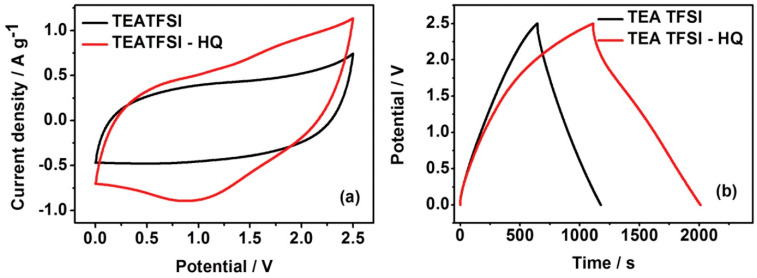
(**a**) Cyclic voltammograms of the SC at 10.0 mV s^−1^; (**b**) galvanostatic charge–discharge at 0.57 mA cm^−2^ in neat [TEA][TFSI] and [TEA][TFSI]/HQ (0.3 M). Reprinted 2015 from [143] with permission from Elsevier.

The electrochemical performance of SCs applied with [TEA][TFSI] mediated with hydroquinone (HQ) was studied and AC was adopted as an electrode for these SCs [93]. The specific capacitance was increased from 42 F g^−1^ to 72 F g^−1^ at 0.57 mA cm^−2^ when 0.3 M HQ was added to [TEA][TFSI]. The pseudo-capacitance contribution of HQ with the faradaic reaction of HQ/Q led to these results (Figure 10).

### 3.3. Ionic Liquid as a Quasi-Solid and All-Solid Electrolyte

**Figure 11:**
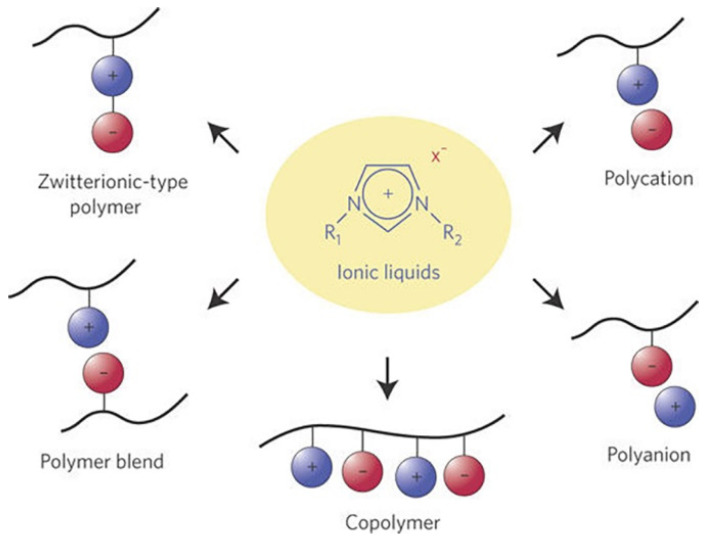
Ionic liquid polymeric electrolyte. Reprinted 2009 from [35] with permission from Springer Nature.

Liquid electrolytes are not desirable for flexible designs because it is necessary to encapsulate them. To solve this issue, numerous studies, such as those that have investigated solidifying the IL, have been widely conducted because ILs retain their ionic conductivity when solidified, whereas organic solvents do not. The ILs with a polymer matrix are typically used to represent solid-state electrolyte combinations. Figure 11 shows various methods for making solid electrolytes using a polymer matrix and ILs.

#### 3.3.1. Ionic-Liquid-Embedded Polymer Electrolyte

The IL-embedded polymer electrolyte can be referred to as an ion gel. Ion gel electrolytes have both a polymer electrolyte and IL [144,145,146]. The IL ratio of ion gels affects ionic conductivity. The more IL ratio of the ion gel embrace, the higher ionic conductivity showed. On the other hand, a high ratio of IL in the ion gel causes weak mechanical stability. Thus, the optimum ratio of the polymer electrolyte and IL must be determined to achieve high ionic conductivity and mechanical stability.

**Figure 12:**
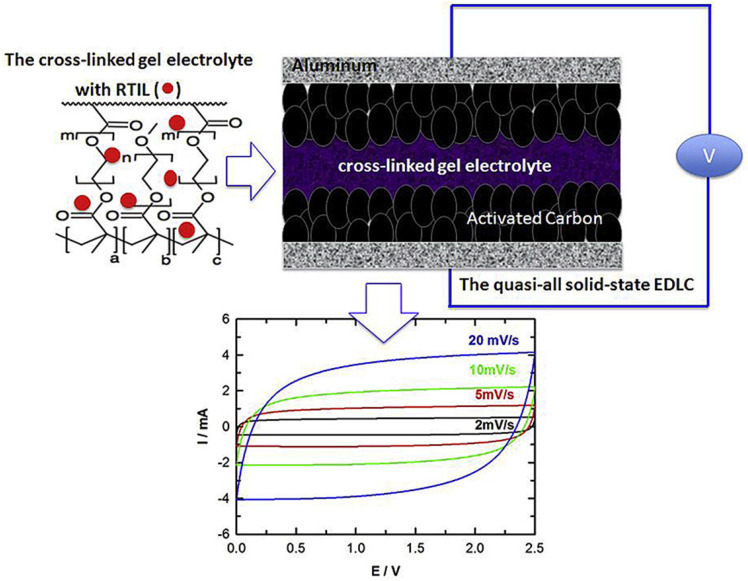
The scheme of the cross-linked gel polymer electrolyte that is composite with methacrylate and dimethacrylate oligomers dissolved in [PMPyrr][TFSI] followed by a cyclic voltammogram depending on scan rates. Reprinted 2011 from [64] with permission from Elsevier.

The prototype ion gel electrolytes consist of ILs, organic solvents, and polymers, so-called gel polymer electrolytes of energy storage devices. In 1997, Fuller et al. reported an ion gel using PVdF-HFP as the polymer matrix [147,148]. The ion gel consisting of [EMI][BF_4_], [EMI][CF_3_SO_3_], and PVdF-HFP achieved an ionic conductivity of 5.8 mS cm^−1^. Ghamouss et al. presented a quasi-solid electrolyte that was combined methacrylate and dimethacrylate oligomers dissolved in [PMPyrr][TFSI] via free radical polarization, as shown in Figure 12. The advantage of this incorporation is that the formed electrolyte can be used as a separator, is leakage-free, and provides a wide electrochemical stability window. The SC that used the prepared electrolyte showed a specific energy density of 16 Wh Kg^−1^, a power density of 1.1 kW kg^−1^, and coulombic efficiency of 99.9%.

**Figure 13:**
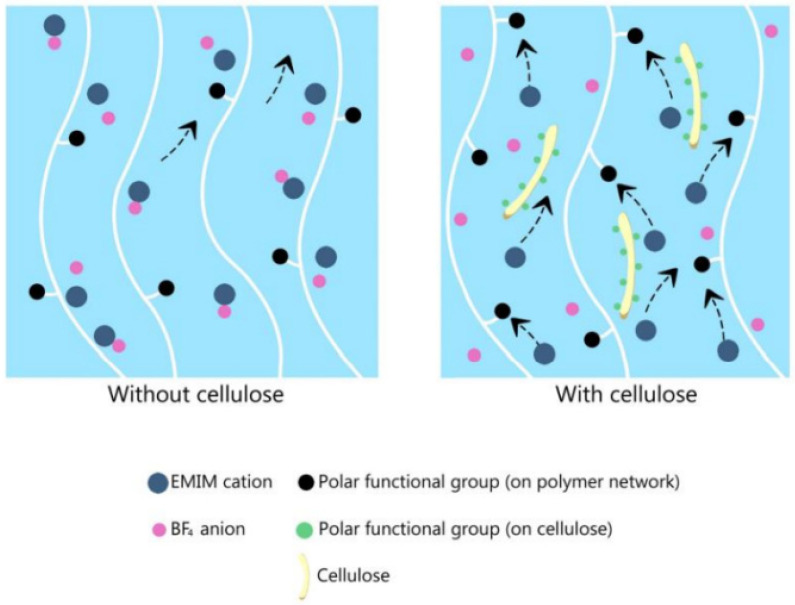
Ion pathway of the ion gel PHEMA-co-PEGDMA/[EMI][BF_4_]. Reprinted 2018 from [94] with permission from the European Chemical Societies.

Most ion gel electrolytes have a relatively lower ionic conductivity than liquid-phase ILs. To overcome this issue, various types of organic or inorganic fillers have been added to the ion gel. Kim et al. synthesized an ion gel with PHEMA-co-PEGDMA/[EMI][BF_4_] and added cellulose as a filler [94]. Figure 13 shows the schematic illustration of the electrolyte. Without cellulose, the ionic conduction path was not sufficiently formed. With cellulose, on the other hand, the hydroxyl group and the IL formed hydrogen bonds in the ion gel. This caused an interaction between the polymer and the IL, resulting in ion-pair dissociation. Furthermore, the polar functional group of the cellulose promoted ion transfer by providing an ionic conduction channel. The maximum ion conductivity value of 12.27 mS cm^−1^ was observed under 3 wt% cellulose fillers. This value was a 266% improvement compared to that of the ion gel electrolyte without cellulose, and it was close to the previously reported [EMI][BF_4_] ionic conductivity (14 mS cm^−1^).

Other ways to increase ionic conductivity were proposed by Liu et al. [149]. High ionic conductivity was achieved by fabricating the aligned ion gel electrolyte. The SCs with the aligned ion gel electrolyte exhibited a 29% higher specific capacitance (176 F g^−1^ at 25 °C and 1 A g^−1^) than SCs with an equivalent non-aligned ion gel electrolyte because of the directional ion pathway.

SCs are considered an attractive power source due to their high power density and extremely long life cycles. However, SCs have the intrinsic problem of low energy density. The energy density is related to the specific capacitance of electrode materials and cell operating voltage. For these reasons, porous electrodes, such as carbonaceous materials, have been investigated due to their high surface area, which increases non-faradaic EDL capacitance. In addition, redox-active materials have also been investigated, as mentioned in a previous chapter. To overcome aqueous medium-induced limiting voltage issues, gel-type redox-active electrolytes are suggested. Recently, Tu et al. prepared a gel polymer electrolyte consisting of [BMI][I], poly(vinyl alcohol) (PVA), and Li_2_SO_4_ [95]. Changes in the salt of the gel polymer electrolyte triggered the redox reaction. The [BMI][I] served as a plasticizer and provided a pseudo-capacitance from the reversible faradaic reactions at the electrode/electrolyte interfaces. Moreover, the tensile strength of the SC significantly increased while its flexibility was maintained. Equation (3) presents the redox pairs in the PVA–Li_2_SO_4_–[BMI][I] gel polymer electrolyte:

(3) $$\begin{array}{c} 3I^{-}↔\;I_{3}^{-}+2e^{-}\\ 2I^{-}↔\;I_{2}+2e^{-}\\ 2I_{3}^{-}↔\;3I_{2}+2e^{-}\\ I_{2}+6H_{2}O↔2{\mathrm{IO}}_{3}^{-}+12H^{+}+10e^{-} \end{array}$$

Furthermore, Yadav et al. used a dual redox-additive material (KI and diphenylamine (DPA)) to maximize the redox reaction at the electrode/electrolyte interface in non-aqueous gel polymer electrolytes with [BMI][TFSI] [146]. The proposed electrolytes showed a wide potential window (6.2 V vs. Ag), high ionic conductivity (σ = ∼0.452 × 10^−2^ S cm^−1^), high flexibility, free-standing properties, and remarkable thermal stability at up to 230 °C. Furthermore, the synergistic effect of the dual redox-additive material results in high specific energy and power densities of about 73.2 Wh kg^−1^ and 34.8 kW kg^−1^, respectively, with an enhanced specific capacitance of 337 F g^−1^.

In relation to increasing the operating voltage, the cell working voltages depend on the electrochemical stability window of the electrolyte. Most of the mentioned ILs and ion gel worked at 2.0–3.5 V. These operating voltages were insufficient to overcome the intrinsic low energy density limitation of SCs hindering their broad application. Thus, ion gel electrolytes that exhibit excellent electrochemical stability have been extensively investigated. Pandey et al. used zeolite as an additive to enhance the working voltage; the zeolite-incorporated SCs were stable up to 4.1 V [43]. A cross-linked polymer matrix was suggested as a solid electrolyte for high-voltage SCs.

**Figure 14:**
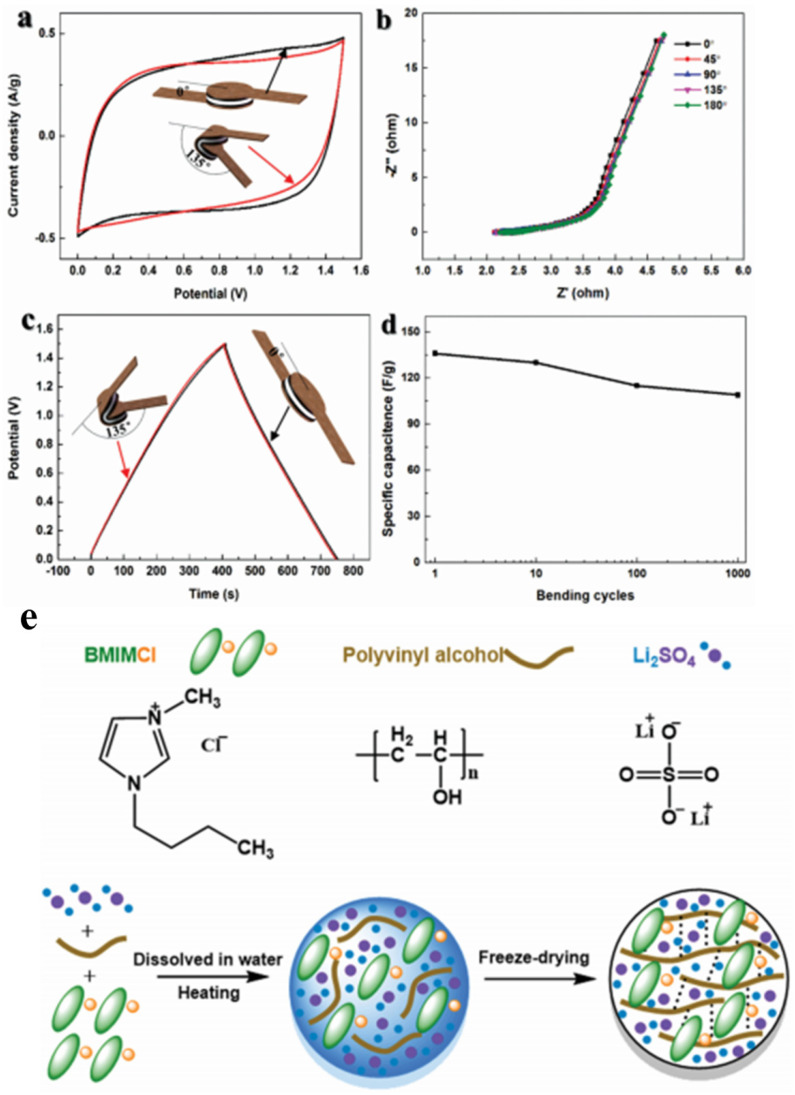
(**a**) Cyclic voltammogram for the SCs of PVA-[BMI][Cl]-Li_2_SO_4_ FGPE with bending test at a scan rate of 10 mV s^−1^; (**b**) Nyquist plot for the SCs of PVA-[BMI][Cl]-Li_2_SO_4_ FGPE at different bending angles; (**c**) galvanostatic charge-discharge for the SCs of PVA-[BMI][Cl]- Li_2_SO_4_ FGPE with bending test at a current density of 0.15 A g^−^^1^; (**d**) variation of specific capacitance after 1000 bending cycles (bending angle = 135°); (**e**) synthesis of PVA-[BMI][Cl]- Li_2_SO_4_ FGPE. Reprinted 2017 from [150] with permission from Advanced Materials Technologies.

The flexibility of the polymer chain is one of its unique characteristics. The soft and hard segments of the polymer backbone enable its flexibility. Flexibility is a necessary feature for wearable devices. Maolin et al. utilized these polymers as the electrolyte in SCs. The gel polymer was mixed with [BMI][Cl], Li_2_SO_4_, and PVA (ratio of 3:2.2:1) using the freeze-drying method [96]. A bending test was conducted on the SC fabricated with the electrolyte and with AC as the electrode, as shown in Figure 14. Although the SCs were bent several times, they had nearly similar cyclic voltammetry and GCD profiles (Figure 14a,c). Similar resistances are observed via the Nyquist plot in the case of different bending angles (Figure 14b). Furthermore, the SCs had a specific capacitance retention of 82% after 1000 bending cycles at 135 °C. These results mean that the proposed SCs exhibited excellent flexibility.

**Figure 15:**
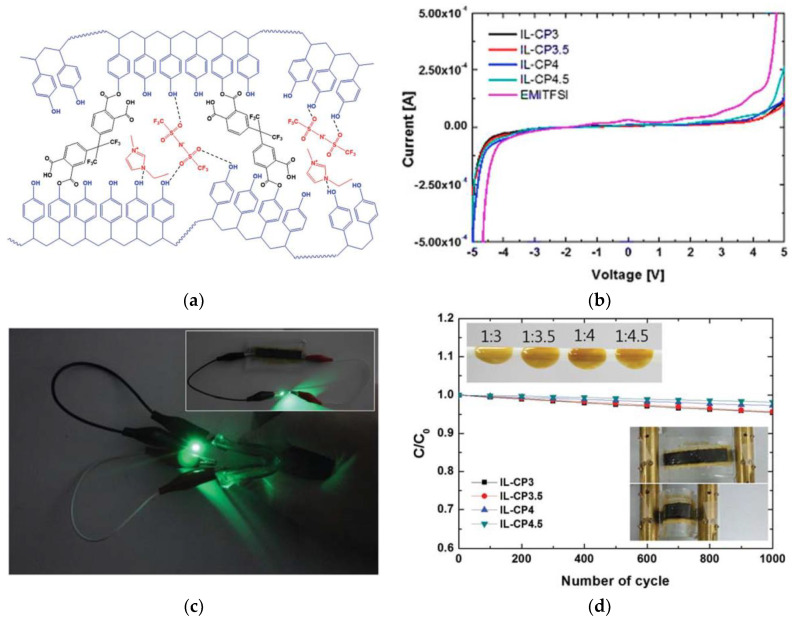
(**a**) Estimated structure of solid electrolyte; (**b**) linear sweep voltammogram for [EMI][TFSI] and solid electrolyte; (**c**) flexibility of solid electrolyte and bending performance of SCs with solid electrolyte for 1000 cycles; (**d**) photograph of a green light-emitting diode (LED) powered by a single SC with a solid electrolyte. Reprinted 2015 from [60] with permission from IOP Publishing.

In 2016, a quasi-solid polymer electrolyte that was operated to 4 V was reported [41]. This electrolyte was composed of a cross-linked (poly-4-vinylphenol (c-P4VPh)) polymer and [EMI][TFSI]. The cross-linked polymer served as a frame, while the IL aided in increasing the ionic conductivity. This electrolyte system improved the electrochemical stability of SCs because the hydrogen bonding between [EMI][TFSI] and c-P4VPh increased the electrochemical stability window while maintaining the quasi-solid state. Figure 15a shows the estimated structure. The electrolyte composite was thermally stable up to 300 °C and electrochemically stable at over 7.0 V (Figure 15b). The SCs with the prepared electrolyte applied demonstrated a specific capacitance of 172.5 F g^−1^ and an energy density of 72.3 Wh kg^−1^. Additionally, the introduced SCs had excellent flexibility, as confirmed by the bending test (Figure 15c,d) [41].

Cho et al. presented an SC which is adopted an ion-gel composed of [EMI][BF_4_] and a polymer matrix of PVdF-HFP as electrolyte. Furthermore, PVdF-HFP/[EMI][BF_4_] achieved an improved specific capacitance of 323 F g^−1^ at 4 V by optimizing the electrochemically active surface of the carbonaceous electrodes. To achieve an electrochemically high performance, this research proposed morphological manipulation for controlling carbon electrode materials to be suitable for IL electrolytes [97].

**Figure 16:**
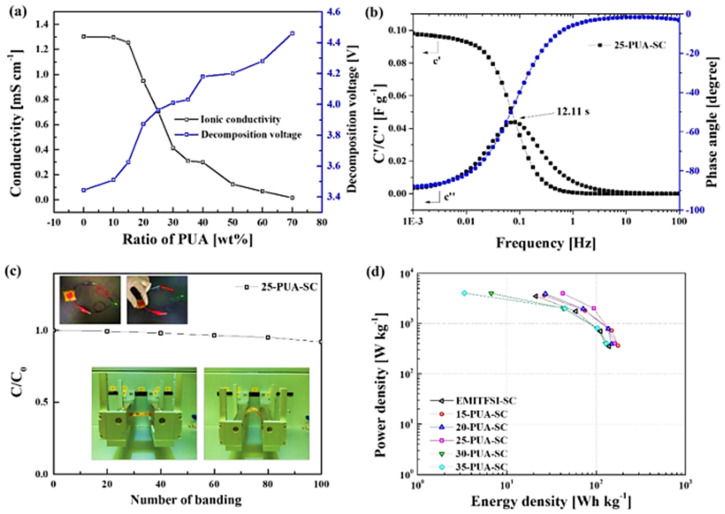
(**a**) Ionic conductivities and decomposition voltages of [EMI][TFSI] and ion gel; (**b**) relaxation time and phase−angle of SC based on ion gel; (**c**) bending test of SC based on ion gel and photographs of a light-emitting diode working at 4 V; (**d**) Ragone plots of SC based on ion gel. Reprinted 2021 from [98] with permission from Elsevier.

Recently, our team proposed a solid electrolyte adopting PUA/[EMI][TFSI] through the novel and facile process while maintaining the free-standing properties and high ionic conductivity [98]. Most reported ion gel electrolyte fabrication methods require additional solvents. However, the proposed ion gel electrolyte was prepared using a solvent-free in situ ultraviolet (UV) polymerization method. The operating voltage of the ion gel is enhanced by the hydrogen bonding between the polymer matrix and anion. The suggested ion gel contains up to 90 wt% of [EMI][TFSI] owing to the high compatibility of the PUA and IL. Additionally, the proposed electrolyte exhibits excellent flexibility without a decrease in ionic conductivity and thermal stability. The SCs, which comprise 25 wt% PUA and 75 wt% [EMI][TFSI], exhibited a specific capacitance of 150.88 F g^−1^ at 0.1 A g^−1^. They also denoted a high specific energy density of 93.93 Wh kg^−1^ at 2000.96 Wh kg^−1^. Capacitance retention of the ion-gel-applied SCs were 99% after 1000 cycles and exhibited remarkable flexibility while maintaining 92% of capacitance retention after 100 times bending (Figure 16).

#### 3.3.2. Poly Ionic Liquid as a Solid Electrolyte

A PIL is a polyelectrolyte that is composed of a polymer skeleton and an IL species in repeating units [151]. The PILs (from oligomers to high-molecular-weight polymers) are affected by the IL properties, including thermal stability, negligible vapor pressure, non-flammability, relatively high ionic conductivity, and broad electrochemical stability window [151,152,153,154]. The structures and properties of PILs have been used for various applications such as electrolytes, electrochemical devices, electrochemical catalyst supports, and porous polymer structures. So far, PILs have been investigated to improve the compatibility of PILs with other components as well as to take advantage of the versatility of PILs and enhance properties [155,156].

There are two methods for synthesizing PILs: (1) the one-step method and (2) the multi-step method [156]. The one-step method involves direct chain-growth polymerization of ILs with or without non-ionic monomers. This method has the advantage of being straightforward and easy to perform. In the multi-step method, PIL composites are generated via the bonding of the precursors of a substance and the IL monomer unit followed by a chemical reaction. There are two different approaches to the multi-step method: (1) the step-growth polymerization of IL monomers and (2) post-modification of polymer chains with IL monomers. In the step-growth polymerization of IL monomers, the IL monomers are first immobilized by substances before being polymerized to generate PILs. This process has the advantage of allowing composites to be designed using a variety of IL monomers and polymerization methods. In the post-modification of polymer chains with IL monomers, the polymer structure is formed first and then IL monomers are bound to the polymer matrix. The sizes or spatial distributions of the substances in the composite are well controlled.

The properties of ILs, such as high thermal stability, wide range of electrochemical stability, and high ionic conductivity are essential in solid electrolytes. Among them, the high ionic conductivity is the most important property when using PILs as a solid-state electrolyte. The ILs and ion gels are capable of moving anions and cations, whereas PILs are usually single-ion conductors. In PILs, the cation or anion is constrained as part of the polymer backbone [151], resulting in an ionic conductivity that is generally lower than that of monomeric ILs. This phenomenon results from an increase in the glass transition temperature (T_g_) and a decrease in the mobile ions after covalent or ionic bonding [155]. The factors that affect the ionic conductivity of PILs are the polymer molecular weight and the chemical characteristics of the polymer backbone.

**Figure 17:**
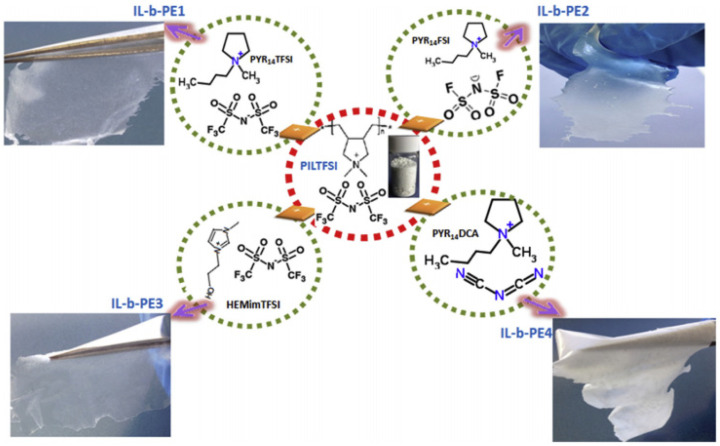
Chemical structure of [PIL][TFSI] and different ILs ([Pyr_14_][TFSI], [Pyr_14_][FSI], [Pyr_14_][DCA], and [HEMI][TFSI] and the respective photo for each membrane. Reprinted 2016 from [99] with permission from Elsevier.

The chemical structure of PILs determines the decomposition temperature, which is related to thermal stability [99]. The chemical characteristics of an anion are one of the factors that affect the decomposition temperature. To investigate the anion effect, Marcilla et al. reported on four IL-based polymer electrolytes (IL-b-PE) (Figure 17). The four IL-b-Pes PEs were synthesized by blending a [PIL][TFSI] with four different ILs: [Pyr_14_][TFSI] (IL-b-PE1), imide [Pyr_14_][FSI] (IL-b-PE2), 1-(2-hydroxyethyl)-3-methylimidazolium bis(trifluoromethylsulfonyl)imide ([HEMI][TFSI]) (IL-b-PE3), and [Pyr_14_][DCA] (IL-b-PE4) (Figure 17) [99]. The physical and chemical properties of the four solid electrolytes are largely determined by the IL properties. The IL-b-PEs containing relatively small anions showed higher ionic conductivity than those containing large anions. In addition, the electrochemically stable pyrrolidinium cation and the IL-b-PEs with TFSI and FSI anions showed a wide electrochemical stability window. The full cell was fabricated using an AC electrode and performed best in IL-b-PE2, which compromised both the ionic conductivity and electrochemical stability window. The energy density, power density, and specific capacitance were as high as 150 F g^−1^, 36 Wh kg^−1^, and 1170 W kg^−1^, respectively, with a 3.5 V electrochemical stability window.

**Figure 18:**
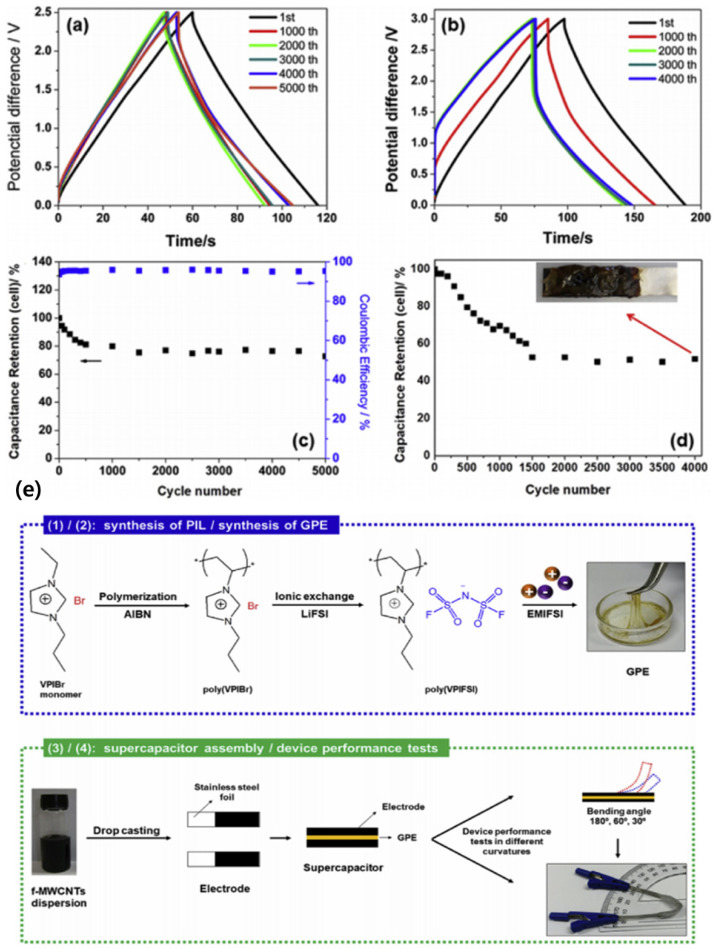
Galvanostatic charge–discharge profile for the SCs at 0.5 A g^−1^ at (**a**) 2.5 V; (**b**) 3.0 V. The cycle stability at (**c**) 2.5 V; (**d**) 3.0 V. (**e**) Schematic illustration of the experimental procedures. Reprinted 2019 from [152] with permission from Elsevier.

In 2019, Lavall et al. suggested combining synthetic PILs composed of poly(1-vinyl-3-propylimidazolium bis(fluorosulfonyl)imide) (poly-VPIFSI) and [EMI][FSI] to create a new gel with adhesion characteristics [152]. The combination of a PIL matrix and ILs resulted in the synthesis of adhesive an GPE with high conductivity at room temperature (Figure 18). The prepared electrolyte had excellent adhesion and the wettability of a gel electrolyte to the electrode surface, and prevented leakage into the PIL matrix, demonstrating excellent PIL–IL interactions and high cycle stability. In addition, when the cell was folded, it had improved kinetics and cycling with minor changes in the capacitance, energy density, and power density.

As mentioned above, PIL electrolytes are single-ion conductors since the cations or anions are confined in the polymer structure. Thus, the ionic conductivity of PILs is comparatively lower than that of IL monomers. To solve these issues, PIL composite electrolytes were investigated, such as PIL-IL and PIL-salt. Oliveira et al. developed gel polymer electrolytes prepared with pyrrolidinium-based [PIL][TFSI]. Additionally, two ILs, [MBI][FSI] and [DPI][TFSI], were used as an ion conductor [100]. The gel electrolytes showed electrochemical stability windows of 2.8 V and 3.0 V, respectively. Moreover, the prepared electrolytes exhibited a conductivity of 3.2 × 10^−3^ S cm^−1^ [MBI][FSI] and 5.0 × 10^−4^ S cm^−1^ [DPI][TFSI] at 25 °C.

## 4. Conclusions and Prospective

This article reviewed research on IL applications, focusing on LIBs, Li-S, and SCs.

With the continuous development of energy storage and conversion systems, ILs have played an important role in energy storage and conversion systems to enhance the electrochemical characteristics, reliability, and safety of these systems.

Conventional electrolytes for LIBs have struggled with humidity sensitivity, the repeated formation of SEI layers, operating voltage limitations, and flammability because of the use of organic solvents. However, ILs are suitable materials for overcoming these problems. ILs have almost no vapor pressure, are flame retardant and thermally stable, and can simultaneously allow free combinations of cations and anions to control the acidity of the electrolyte. Furthermore, ILs can provide a high operating voltage because ILs have a wide electrochemical stability window of up to 6 V. Since this value is over 30% higher than the existing operating voltage of 4.5 V, it can greatly contribute to the improvement of the energy density of LIBs.

ILs have been used as an electrolyte for LIBs in various methods: (i) organic solvents have been replaced with ILs to reduce volatility and flammability, (ii) ILs have been mixed with conventional organic electrolytes to suppress electrolyte degradation due to stable SEI layer formation, thereby improving lithium storage performance, (iii) ILs have been used to minimize polysulfide dissolution, and (iv) ILs have been used with PEO and PVdF-HFP to improve the low conductivity of solid electrolytes.

ILs are also used as an electrolyte for SCs. In particular, when using ILs as electrolytes, the addition of salts is not required, and similar to LIBs the operating voltage can be greatly increased.

ILs have also been used as an electrolyte for SCs in various methods: (i) the high electrochemical potential window (over 3.5 V) of ILs has been used to increase energy density, (ii) the anions of two ILs can be mixed to form eutectic ILs, (iii) according to the ion size of the ILs and the pore size adjustment of the carbon electrode, ILs contribute to the characteristics of EDLs, (iv) ion gels made of ILs can be used as a solid electrolyte in wearable devices to obtain high energy density, and (v) PILs can be used to increase energy density and long-term cycle stability.

The application of ILs to energy storage devices has been continuously conducted, and it is expected to continue in the future to improve the electrochemical performance and stability of energy storage devices. However, the price of ILs is ~1000 dollars per kg based on imidazolium salt, which is expensive compared to conventional solvents, so research on reducing the cost of producing ILs is inevitable. In addition, more studies on the compatibility of LIB and SC electrodes with IL electrolytes and methods to improve the ionic conductivity of IL electrolytes are necessary. Furthermore, research on new combinations of ILs or eutectic salts that combine two or more types of ILs is also required. In addition, as studies on ILs progress, it is expected that ILs can be applied to future energy storage and conversion devices, such as multivalent ion batteries and metal air batteries, in addition to LIBs and SCs introduced in this paper.

In this regard, we believe that this paper can inspire researchers to pursue more advanced applications of ILs in energy storage devices.

## Figures and Tables

**Table 1:** The lists of the representative ILs applied in LIBs and SCs as electrolytes.

Classification	Acronyms	Ionic Liquid	Reference(s)
Li-ion batteries	[PMI][TFSI]	1-propyl-3-methylimidazolium bis(trifluoromethylsulfonyl)imide	[70]
	[BMI][TFSI]	1-Butyl-3-methylimidazolium bis(trifluoromethylsulfonyl)imide	[70]
	[EMI][TFSI]	1-ethyl-2,3trimethyleneimidazolium bis(trifluoromethane sulfonyl)imide	[72]
	[PP_13_][TFSI]	N-methyl-N-propylpiperidinium Bis(trifluoromethanesulfonyl)imide	[73]
	[Pyr_14_][DCA]	N-butyl-N-methyl pyrrolidinium-dicyanamide	[74]
	[Pyr_14_][TFSI]	N-butyl-N-methyl pyrrolidinium bis(trifluoromethylsulfonyl)imide	[74]
	[Pyr_14_][TFSAM]	N-butyl-N-methyl pyrrolidinium bis(trifluoromethylsulfonyl)-N-cyanoamide	[74]
	[N_2_(2o1)_3_][TFSI]	N-ethyl-N,N,N-tri-(2-methoxyethyl)ammonium bis(trifluoromethanesulfonyl)imide	[75]
	[(N_2_(2o1)_2_(2o2)][TFSI]	N-ethyl-N,N-di-(2-methoxyethyl)-N-2-ethoxyethylammonium bis(trifluoromethanesulfonyl)imide	[75]
	[(N_3_(2o1)_3_)][TFSI]	N-propyl-N,N,N-tri-(2-methoxyethyl)ammonium bis(trifluoromethanesulfonyl)imide	[75]
	[(N_4_(2o1)_3_)][TFSI]	N-butyl-N,N,N-tri-(2-methoxyethyl)ammonium bis(trifluoromethanesulfonyl)imide	[75]
	[C_4_mpyr][TFSI]	N-methyl-N-butyl-pyrrolidinium bis(trifluoromethanesulfonyl)imide	[76]
	[PfM_pyr_][FSI]	1-Methyl-1-propyl-3-fluoropyrrolidinium bis(fluorosulfonyl)-imide	[77]
	[C_3_mpyr][FSI]	N-methyl-N-propyl-pyrrolidinium (fluorosulfonyl) imide	[78]
LiS batteries	[PP_13_][TFSI]	N-methyl-N-propylpiperidinium bis(trifluoromethanesulfonyl)imide	[79]
	[LiG_3_][TFSI]	Li(triglyme) bis(trifluoromethylsulfonyl)imide	[80]
	PEO, LiTFSI-[TBP][HP]	poly(ethyleneoxide)-lithium bis(trifluoromethylsulfonyl)imide-tetrabutylphosphonium 2-hydroxypyridine	[81]
	PVdF-HFP/PMMA/[BMI][BF_4_]	(poly(vinylidene fluoride-co-hexafluoropropylene)/poly(methyl methacrylate)- 1-butyl-3-methylimidazolium tetrafluoroborate))	[82]
	PVdF-HFP/[EMI][DCA]	(poly(vinylidene fluoride-co-hexafluoropropylene)/1-ethyl-3-methylimidazolium dicyanamide))	[83]
	PVdF-HFP/[EMI][TFSI]/rGO-PEG-NH_2_	(poly(vinylidene fluoride-co-hexafluoropropylene)/ (covalent linked 2,2″-(ethylenedioxy) bis (ethylamine) to reduced graphene oxide))	[84]
Supercapacitors	[EMI][BF_4_]	1-ethyl-3-methylimidazolium tetrafluoroborate	[85]
	[EMI][FSI]	1-ethyl-3-methyleneimidazolium (fluorosulfonyl) imide	[86]
	[Pyr_14_][TFSI]	N-butyl-N-methyl pyrrolidinium bis(trifluoromethylsulfonyl)imide	[87]
	[EMI][TFSI]	1-ethyl-3-methyleneimidazolium bis(trifluoromethane sulfonyl)imide	[88]
	[Pyr_14_][FSI]	N-ethyl-N-methylpyrrolidinium (fluorosulfonyl) imide	[89]
	[PIP_13_][FSI]	N-methyl-N-propylpiperidinium (fluorosulfonyl)imide	[89]
	[Pyr][TFSI]	N-butyl-N-methyl pyrrolidinium bis(trifluoromethylsulfonyl)imide	[90]
	[PMP_yrr_][TFSI]	1-Methyl-1propylpyrrolidinium bis(trifluoromeyhanesulfonyl)imide	[91]
	[BTM][TFSI]	Butyltrimethylammonium- bis(trifluoromethylsulfonyl)imide	[92]
	[TEA][TFSI]	Trimethylamine- bis(trifluoromethylsulfonyl)imide	[93]
Quasi-solid-state supercapacitors	PHEMA-co-PEGDMA/[EMI][BF_4_]	poly(2-hydroxyethyl methacrylate) and poly(ethylene glycol) diacrylate/1-ethyl-3-methylimidazolium tetrafluoroborate	[94]
	[BMI][I]	1-butyl-3-methylimidazolium iodide	[95]
	[BMI][Cl]	1-butyl-3-methylimidazolium chloride	[96]
	PVdF-HFP/[EMI][BF_4_]	(poly(vinylidene fluoride-co-hexafluoropropylene) /- 1-ethyl-2,3methylimidazolium tetrafluoroborate))	[97]
	PUA/[EMI][TFSI]	(polyurethane acrylate-(1-ethyl-3-methylimidazolium- bis(trifluoromethylsulfonyl)imide)	[98]
All-solid-state supercapacitors	[Pyr_14_][TFSI]	1-butyl-1-methylpyrrolidinium bis (trifluoromethanesulfonyl)) imide	[99]
	[Pyr_14_][DCA]	1-butyl-1-methylpyrrolidinium dish Amide	[99]
	[PIL][TFSI]	poly(diallyldi-methylammonium) bis (trifluoromethylsulfonyl) imide	[100]
	[EMI][FSI]	1-ethyl-3-methyleneimidazolium (fluorosulfonyl) imide	[100]
	[MBI][FSI]	1-methyl-3-butylimidazolium (fluorosulfonyl) imide	[100]
	[DPI][TFSI]	1,2-dimethyl-3-propylimidazolium bis(tri-fluoromethylsulfonyl) imide	[100]

**Table 2:** The properties of representative ILs.

Type of Cation	Ionic Liquid	Melting Point (°C)	Density (g mL^−1^) at 25 °C (lit.)	Cnductivity (mS cm^−1^)	Electrochemical Stability Window (V)	Reference(s)
Imidazolium	[EMI][BF_4_]	15	1.294	13–15	4 (ref. elecrode: carbon)	[101,102]
	[EMI][TFSI]	−15	1.53	8–10	3.5–3.7 (ref. elecrode: carbon)	[91]
	[BMI][TFSI]	1	1.44	3.9	4.5–5 (ref. elecrode: carbon)	[70]
Pyrrolidinum	[Pyr_14_][TFSI]	−6	1.4216	2.5–3	3.5 (ref. elecrode: carbon)	[103]
	[Pyr_14_][DCA]	−55	0.95	10.8	3 (ref. elecrode: carbon)	[104]
Piperidinium	[PP_13_][TFSI]	12	151	1.4	5–6 (ref. electrode: Li)	[73]
	[PP_13_][FSI]		95	3.7	5–6 (ref. electrode: Li)	[73]

**Table 3:** CDC porosity measurements using the Ar gas sorption technique. Reprinted 2008 from [88] with permission from the American Chemical Society.

Chlorination Temperature ( °C)	BET SSA (m^2^ g^−1^)	Pore Volume (cc g^−1^)	Average Pore Widt (nm)	Maximum Pore Width ^a^ (nm)
400	1113	0.51	0.65	1.12
500	1140	0.50	0.68	1.18
550	1202	0.51	0.72	1.29
600	1269	0.60	0.74	1.23
700	1401	0.66	0.76	1.41
800	1595	0.79	0.81	1.54
1000	1625	0.81	1.10	2.80

^a^ 85% of pore volume is below this size.

## Data Availability

No new data were created or analyzed in this study.
